# Gastric emptying and duodenal motility upon intake of a liquid meal with monosodium glutamate in healthy subjects

**DOI:** 10.1002/phy2.187

**Published:** 2014-01-06

**Authors:** Hidemi Teramoto, Toshiyasu Shimizu, Hideto Yogo, Yuuta Nishimiya, Shinji Hori, Takashi Kosugi, Shinsuke Nakayama

**Affiliations:** 1 Department of Internal Medicine, Kojin Hospital, Nagoya, 463‐8503, Japan; 2 R'Tech Corporation, Hamamatsu, 431‐2103, Japan; 3 Department of Radiology, Kojin Hospital, Nagoya, 463‐8503, Japan; 4 Department of Cell Physiology, Nagoya University Graduate School of Medicine, Nagoya, 466‐8550, Japan

**Keywords:** Gravity center, image stabilization, motion velocity, numerical evaluation, rapid MRI

## Abstract

Glutamate is thought to serve as a special signal for gut functions. We investigated the effects of monosodium l‐glutamate (MSG) on gastric emptying and duodenal motility. Ten healthy male volunteers underwent rapid magnetic resonance imaging (MRI) of the abdomen. Coronal images were successively acquired after ingestion of liquid meal (200 kcal in 200 mL: 9 g protein, 28.4 g carbohydrate, 5.6 g fat, 370 mg Na^+^) with and without 0.5% MSG. During the acquisition of MRI, participants breathed freely. In all participants, the gastric residual volume gradually decreased to 80.1 ± 14.2% without MSG and to 75.9 ± 14.3% with MSG after 60 min (*P *= 0.45 between the groups, *n* = 10). In two of 10 participants, gastric emptying slowed down significantly, whereas in the remaining eight participants, gastric residual volume decreased to 84.0 ± 13.1% without MSG, and to 73.0 ± 14.6% with MSG after 60 min (*P* = 0.015, *n* = 8). There was no difference in the shape of the stomach between groups. In four of the eight participants responding positively to MSG, the duodenum wall was sufficiently identified to quantify the motions. The inclusion of MSG enhanced duodenal motility, judging from changes in (1) the magnitude of the duodenal area, (2) the center of gravity, and (3) the mean velocity of the wall motions. The third parameter most significantly indicated the excitatory effect of l‐glutamate on duodenum motility (~ three‐ to sevenfold increase during 60 min, *P *< 0.05, *n* = 4). These results suggest that MSG accelerates gastric emptying by facilitating duodenal motility, at least in subjects with positive responses to MSG.

## Abstract (precis)

The gut and brain share many neurotransmitters and hormones, thereby gut motility frequently reflects mental conditions in humans. So far, procedures to quantify gut motility in vivo have not yet been established. This study of abdominal rapid magnetic resonance imaging (MRI) demonstrates that monosodium L‐glutamate (MSG) accelerates gastric emptying by facilitating duodenal motility, at least in subjects with positive responses to MSG.

## Introduction

Glutamate plays a signaling molecule in multiple cellular systems in the body. Namely, glutamate is the major excitatory neurotransmitter in the central nervous system, and is most frequently used in normal brain functions. Also, in the oral cavity, glutamate acts as umami taste, one of the five basic tastes together with sweet, sour, salty, and bitter. Upon food intake, the addition of this nonessential amino acid enriches savory and promotes appetite (Uneyama et al. 2009).

In the mucous membrane of the gastrointestinal (GI) tract amino acids serve as signaling molecules (Niijima 1991); Niijima and Meguid 1995); Uneyama 2011). In the stomach, proteolytic enzymes digest protein toward peptide, and the gastric mucous membrane contains only glutamate receptors, including metabotropic glutamate receptors (mGluR). Thus, upon the intake of food, glutamate alone, if contained, facilitates vagal efferent activity in the stomach. On the other hand, in the small intestine, peptides are further digested into individual amino acids to be absorbed, and the mucous membrane possesses numerous receptors for amino acids. Thus, numerous amino acids equally affect vagal efferent activity. These facts imply a special role of glutamate in locally regulating physiological functions in the upper GI tract. In healthy volunteers, ^13^C breath tests have demonstrated that administration of monosodium l‐glutamate (MSG) accelerates gastric emptying upon intake of a protein‐rich meal (Zai et al. 2009). However, so far, how glutamate modulates GI motility and antroduodenal transport has not yet been assessed.

Several techniques are employed for this purpose, and magnetic resonance imaging (MRI) appears to be advantageous among them (Schwizer et al. 1992); Baba et al. 2009); Teramoto et al. 2009). Ultrasonographic evaluation of gut motility frequently depends on personal skill. Scintigraphy and electrogastrography detect gastric motion indirectly (Misu et al. 1990); Stacher et al. 1990). Moreover, radiography requires contrast medium which affects gut motility, and is limited by the exposure time, thus it is unsuitable to monitor slow contractions. On the other hand, MRI provides a noninvasive display of the GI tract due to differences in proton (^1^H) relaxation time among cellular tissues and luminal contents. Recent advances in active shield gradients further enable the reasonably rapid scan of the abdominal region at ~1‐sec intervals (Schwizer et al. 1996); Faas et al. 2001). In addition, compared with other techniques, MRI provides a rather large field of view (FOV), which enables a wide range of the GI tract to be monitored, and to assess cooperative activity of the stomach and the duodenum (Teramoto et al. 2009).

Recently, we have developed in‐house‐made software, which enables numerical evaluation of GI motility in cine MRI, and displayed the association between gastric emptying and duodenal motility upon the ingestion of a liquid meal (Teramoto et al. 2009). In this study, we thus applied the procedures of MRI numerical evaluation to assess the effects of glutamate. We acquired a series of abdominal coronal images in healthy volunteers after ingestion of a liquid meal with and without MSG. The rapid MRI measurements and image analyses revealed enhanced duodenal motility in the subjects who had gastric emptying accelerated in response to glutamate.

## Material and Methods

### Participants

Ten healthy male volunteers (mean age 47.3: 45–50 years; body mass index 23.8 kg/m^2^) participated to the measurement of abdominal rapid MRI. The medical ethics committee of Kojin Hospital (Nagoya, Japan) approved the study protocol, and written, informed consent was obtained from each participant. This study complies with the Helsinki Declaration.

### Magnetic resonance imaging

**Figure 1.**
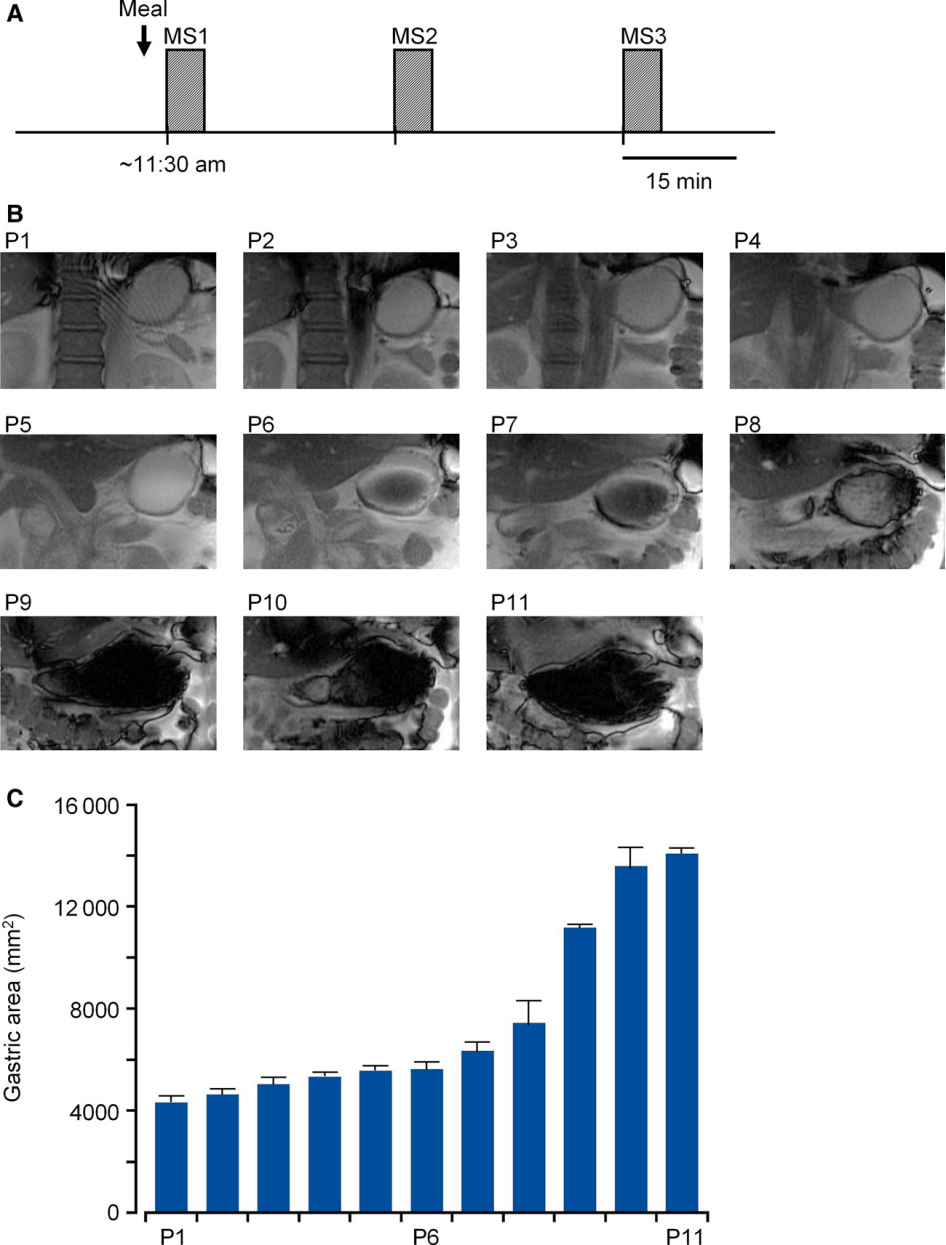
Motility scan (MS) over 11 coronal planes. (A) Study protocol: A series of two‐dimensional rapid MS along 11 coronal planes (P1 to P11, from the dorsal to ventral side with an increment of 9 mm) was repeated three times (MS1, MS2, and MS3) at regular intervals of 30 min immediately after ingestion of liquid meal (~11:15 am). Forty images were acquired in each plane, and therefore a total of 440 images were acquired in each MS. (B) A set of segment images obtained from a participant in MS1. Eleven segment images display the gastric areas at P1–P11. Each segment image was extracted from the initial coronal image acquired in the same plane. For example, the panel in P2 represents the gastric area in the 41st image of the 440 images in MS1. The gastric fundus contains liquid meal from P1 to P8. (C) The magnitude of the gastric area (means ± SD in mm^2^) is plotted against plane number, based on the MS1 scans shown in (B).

MR abdominal imaging was performed on a 3.0‐T horizon MRI system with an echo‐speed gradient (Signa EXCITE HD3.0T; GE Healthcare Japan, Hino, Japan), as described previously (Teramoto et al. 2009), 2012). The localization of the GI tract was measured in the transverse, sagittal, and coronal planes, using a Fast Gradient Echo (GRE) sequence: localization scan (15 images in 12 sec). Then, GI motility was monitored using a fast imaging employing steady‐state acquisition (FIESTA) sequence with a FOV of 440 × 440 mm: motility scan (MS), and a scan resolution was Freq 192 × Phase 288 pixels. The pulse sequence parameters used in FIESTA were as follows: flip angle, 45°; repetition time (TR), 3.9 msec; echo time (TE), 1.7 msec; bandwidth (BW), 125 kHz; slice thickness, 8 mm. Forty images were acquired at 656‐msec intervals on the coronal plane. The same successive coronal image acquisition was repeated in 11 planes, changing the height from the dorsal to the ventral side with an increment of 9 mm per plane (P1–P11 in Fig. 1). In total, 440 sequential images were acquired in each MS, taking ~5 min.

### Study design

All MRI examinations started at around 11:15 am in order to minimize the influence of the circadian rhythm. Participants fasted for ~14 h prior to the examination. After the localization scan was performed to decide the height of 11 coronal planes to be scanned, a series of MSs in these 11 planes was repeated three times at regular intervals of 30 min after the ingestion of liquid meal (200 mL). The initial MS (MS1) was performed immediately after ingestion (Fig. 1A). Subsequently, two series of MS (MS2 and MS3) were performed 30 and 60 min after ingestion, respectively. The whole examination took ~80 min. During MRI acquisitions, participants could breathe freely, and no gated methods were applied. In each participant, two sets of three MS series (MS1, MS2, and MS3) for the ingestion of liquid meal with and without MSG were performed on separate days, with an interval of at least 1 week between series.

The liquid meal (MEDIEF; Ajinomoto Pharma, Tokyo, Japan) contained 200 kcal in 200 mL: 9 g protein, 28.4 g carbohydrate, 5.6 g fat, 370 mg Na^+^, and was taken orally at room temperature (25°C) at a rate of ~200 mL/min. One gram of commercial “Ajinomoto” powder (98% MSG, 2% disodium 5‐ribonucleotide [1% inosinate and 1% guanylate; Ajinomoto Pharma]) was added to 200 mL of liquid meal (Zai et al. 2009). The influence of umami taste perception of l‐glutamate in the oral cavity was minimized by swallowing liquid meal through a straw in a single swallow.

### Data analysis

Motility scan images in the coronal plane in Digital imaging and Communication in Medicine (DICOM) format were imported into in‐house‐made segment software (DigestDyna: R'Tech, Hamamatsu, Japan) (Teramoto et al. 2012). In this software, respiratory motions were cancelled automatically using the following energy function (*E*) as a criterion to be minimized:
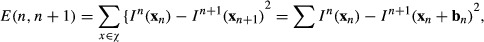
where *I*^*n*^(**x**_*n*_) is the intensity at point **x**_*n*_ = (**x**_*n*_, **y**_*n*_) in the nth image. **b**_*n*_ is the shift vector between the nth and *n* + 1th images. *χ* represents the region of interest (ROI). The shift (**b**_*n*_) in each image was estimated by minimizing *E*(*n*,* n* + 1). This procedure is essentially similar to image stabilization for digital cameras (Morimoto and Chellappa 1996); Yu et al. 2006). It takes ~20 sec to process (stabilize) the ROI of 100 × 200 pixels in 40 images, using a personal computer.

**Figure 2.**
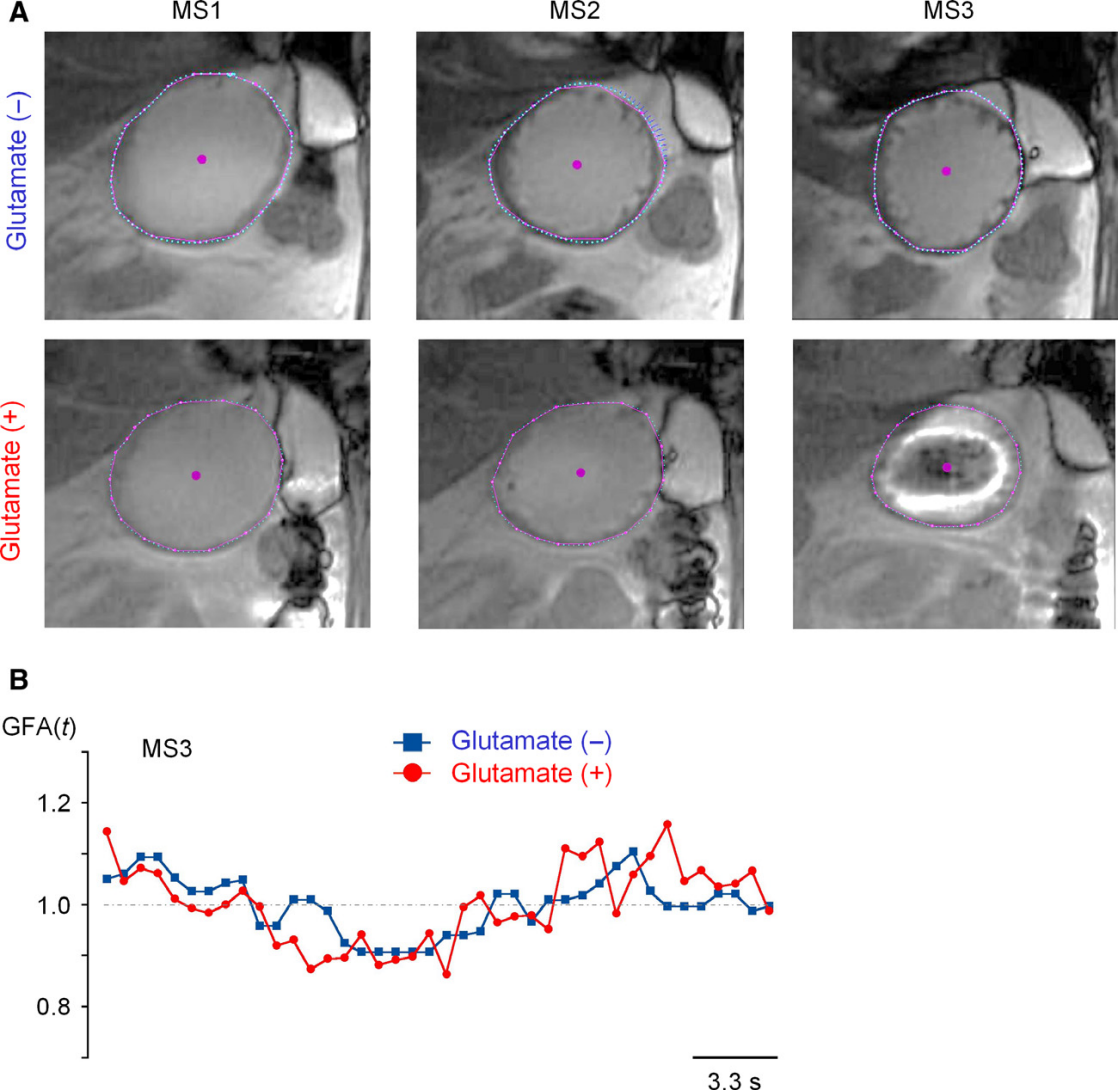
The effect of MSG on gastric emptying observed in a participant indicated by the third asterisk in Figure 5. (A) Coronal segment images of the gastric fundus (P4) after ingestion of liquid meal with and without 0.5% MSG. (B) The motions of gastric fundus represented by the Y‐shift in the center of gravity in 40 successive images in MS3.

To evaluate gastric emptying at a high resolution, the sum of the gastric fundus area in coronal slice planes containing liquid meal (the first 4 planes in Fig. 1B: P1–P4), was used as an index of gastric residual volume. In the ROI of each image (see Fig. 2A), the approximate shape of the gastric fundus was input manually with 20 points (pink), and then the serosal (outer) end of the gastric fundus was defined with 100 points (pale blue) within the same distance by using a spline interpolation procedure. In each coronal plane, the gastric fundus area containing liquid meal was estimated from 40 images acquired successively, averaging the changes in the fundus area due to spontaneous contraction. In each participant, the sum of the gastric fundus area containing liquid meal was estimated at 0, 30, and 60 min, and was normalized relative to that at 0 min.

To rule out possible mechanical disturbance of gastric emptying, the shape and position of the stomach was assessed in all 11 coronal planes. As the gastric wall repeats contractions and relaxations spontaneously, its shape was estimated by averaging 40 images. The shape and position of the stomach did not systematically differ between groups with and without accelerated gastric emptying in response to glutamate (0.5% MSG in 200 mL liquid meal).

**Figure 3.**
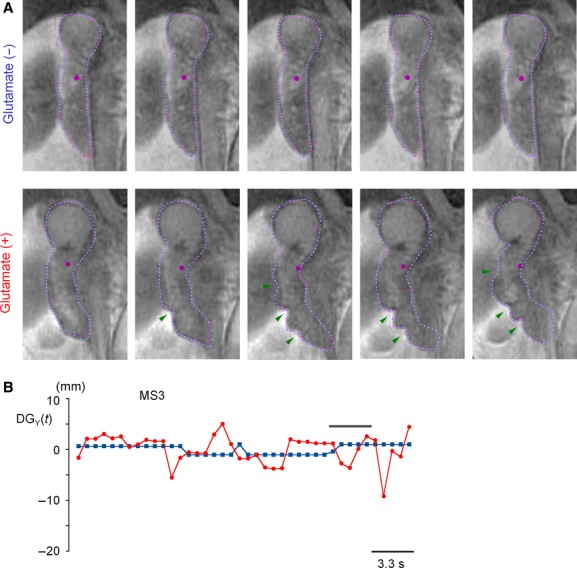
The effect of MSG on duodenal motility observed in the same participant shown in Figure 2. (A) Coronal segment images of the duodenum after ingestion of liquid meal with (upper) and without 0.5% MSG (lower). In upper panels, in order to notify small changes, red and blue lines are added indicating shifts toward the duodenum wall in the next and previous images, respectively. For clarity, line decorations are not used in lower panels which show large contractile activity. Instead, contractile rings are indicated by a green arrow head. (B) Changes in the Y‐shift of the center of gravity of the duodenal area [DG_Y_(*t*)] in the successive 40 segment images in MS3.

To evaluate duodenal motility, a coronal slice showing the Pars superior and Pars descendens rounding the Caput of the pancreas was used. Only one coronal slice plane was available in the present rapid MRI scan with an increment of 9 mm. The approximate shape of the duodenal area was manually input with 30 points, and then the duodenum wall was automatically defined with 100 points of the same distance (pink and blue points in Fig. 3A). As indexes of duodenal motility, three parameters were used: (1) standard deviation of the mean in the duodenal area; (2) total shifts of the center of gravity of the duodenal area; and (3) mean velocity of duodenum wall motions along the normal line (*V*_N_) and tangential line (*V*_T_). Our previous study (Teramoto et al. 2009) showed that these three parameters increase as gastric emptying is facilitated, especially upon administration of a dopamine D_2_ receptor antagonist.

**Figure 4.**
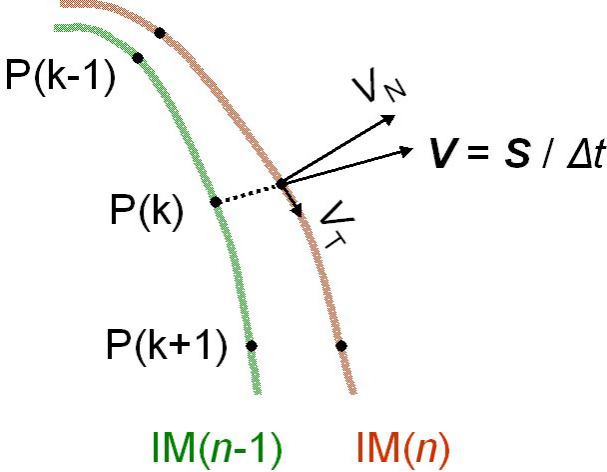
(A) scheme showing procedures used for estimating the magnitude of duodenal wall motions along normal and tangential lines. Green and brown lines represent duodenal walls observed in (*n*‐1)th and (*n*)th segment images (IM(*n*‐1) and IM(*n*)), respectively. In kth point (P(*k*)), the shift between IM(*n*‐1) and IM(*n*) was converted to a velocity vector (*V*) using the interval (*Δt*), and divided into normal and tangential vectors (*V*_N_ and *V*_T_) on the duodenal wall in IM(*n*).

The duodenal area was also estimated from the outer (serosal) surface, as was performed for the area of the gastric fundus. *V*_N_ and *V*_T_ in each image were estimated by dividing the duodenal wall into *n* (= 100) points:
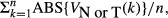


where *V*_N_(*k*) and *V*_T_(*k*) represent the velocity along the normal and tangential line at the *k*th point of the duodenum wall, respectively (Fig. 4). During the calculation of *V*_N_(*k*) and *V*_T_(*k*), the inward and counterclockwise directions of the duodenum wall were assigned a positive value. It is considered that the sum of the absolute velocities at all points of the wall accurately evaluate duodenal motility even when extensions and contractions occur in the same image, being superior to the evaluation procedures using the magnitude of the duodenal area.

Numerical data are expressed as means ± standard deviation. Differences between means were evaluated by paired *t*‐tests. *P* < 0.05 was considered statistically significant.

## Results

### Rapid MRI scans in the gastric region

Ten healthy male volunteers (mean age 47.3) participated in this study. Figure 1 shows the protocol of a rapid MRI scan in the abdomen and an example of a set of stomach segments in a healthy subject. Immediately after ingestion of a liquid meal (200 kcal in 200 mL), a series of two‐dimensional rapid MSs (MS) with a FOV of 440 × 440 mm was applied along 11 coronal planes (P1‐P11). In each coronal plane, 40 images were successively acquired for ~26 sec. Subsequently, the same series of MS was repeated after 30 and 60 min (MS2 and MS3, respectively). During the MRI measurements, participants breathed freely, and respiratory motions were cancelled by a stabilization procedure of segment software.

Segment images in Figure 1B show the whole stomach of a subject measured in 11 planes in MS1. Due to spontaneous contraction, the gastric area changed during the acquisition of 40 images in the same plane. In Figure 1C, the magnitude of the gastric area is shown as the mean and SD. To rule out possible mechanical disturbance of gastric emptying, the shape and position of the stomach was assessed in all 11 coronal planes. Among the 10 participants, no significant difference in shape and position of the stomach, such as gastroptosis, was observed.

### Individual responses to MSG in gastric emptying

During the MRI scan, participants were in the spline position. Thus, the gastric fundus volume estimated from four coronal planes (P1–P4) represented the gastric residual volume (for details, see Methods). The gastric residual volume gradually decreased in the following 60 min (*P *< 0.01 in MS3 vs. MS1, *n* = 10), irrespective of the addition of glutamate (0.5% MSG in 200 mL liquid meal), although the effects of glutamate on the rate of gastric emptying varied.

**Figure 5.**
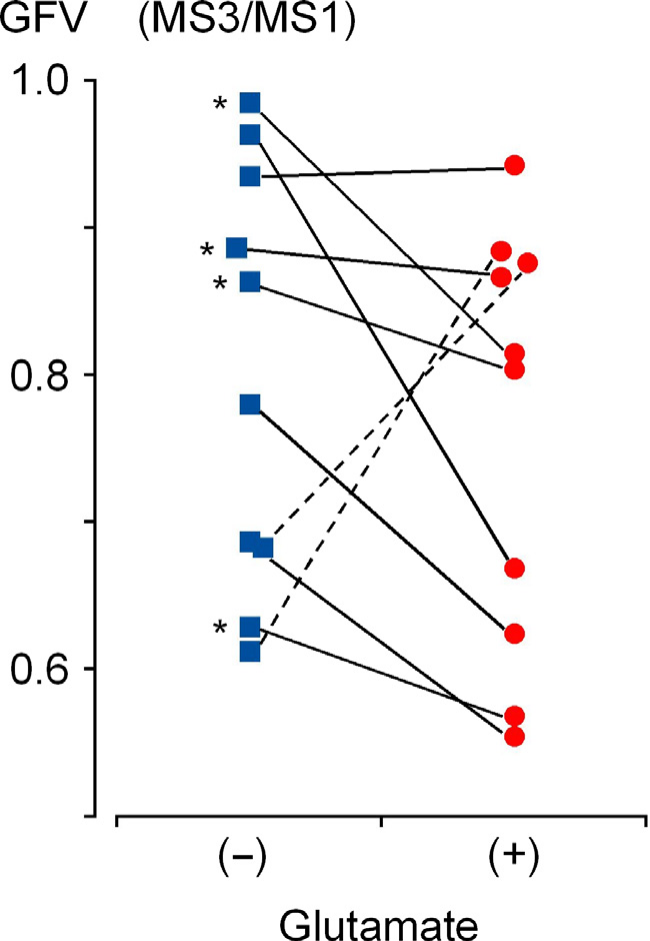
Individual comparison of the effect of MSG on gastric emptying after 60 min (MS3). The gastric fundus volume estimated from segment images acquired in P1–P4 during MS3 was normalized by that in MS1. Two subjects with apparently suppressed gastric emptying are indicated by dotted lines. Asterisks indicate subjects with a duodenal wall sufficiently identified for motility analyses.

**Figure 6.**
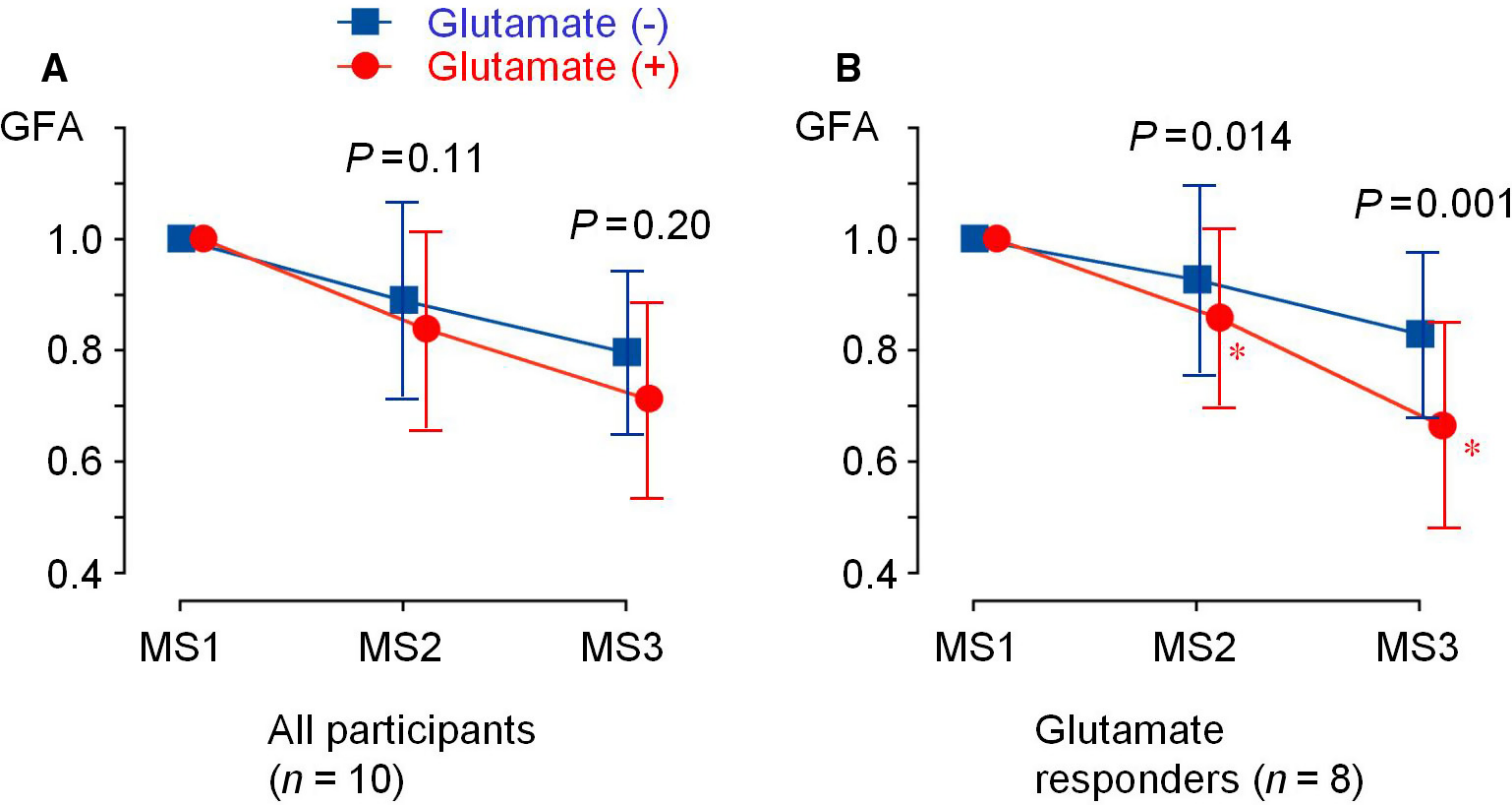
Changes in the gastric fundal area (GFA) estimated from 40 successive images in P4. GFA in MS1 and MS2 are normalized with that in MS1. (A) The statistics indicating all 10 participants. (B) The statistics of eight participants excluding two with negative responses to MSG in gastric emptying. Each *P* value indicates a statistical significance between glutamate (+) and (−).

Figure 5 compares the effects of glutamate on gastric emptying among individuals, using the ratio of the gastric residual volume estimated after 60 min (MS3) to that immediately after ingestion of liquid meal (MS1). In all participants, the gastric residual volume decreased to 80.1 ± 14.2% without MSG and to 75.9 ± 14.3% with MSG after 60 min. However, there was no significant difference between groups (*P* = 0.45, *n* = 10). In two of 10 participants, MSG reduced the rate of gastric emptying (dotted lines in Fig. 5). On the other hand, in the remaining eight participants, the gastric residual volume decreased to 84.0 ± 13.1% without MSG, and to 73.0 ± 14.6% with MSG after 60 min: MSG significantly accelerated gastric emptying (*P* = 0.015, *n* = 8) (see also Fig. 6 summarizing changes in the gastric fundal area in P4). In order to investigate the possible modulation of gut cooperative activity underlying l‐glutamate‐induced acceleration of gastric emptying, we analyzed MRI data obtained from the eight participants with positive responses.

Figure 2 shows a typical accelerating response to MSG on gastric emptying observed in participants (corresponding to the third asterisk from the top in Fig. 5). Segment images of the gastric fundus region after ingestion of liquid meal with (lower) and without the addition of MSG (upper) are compared (A) (see also Videos S1–S4).

In order to assess whether glutamate affects contractility in the gastric fundal area, the time courses of changes in the gastric fundal area with and without MSG are compared in B. Each line shows the gastric fundal area in the P4 plane in 40 successive segment images acquired after 60 min (MS3). The gastric fundal area changes slowly, presumably reflecting the stomach's period of pacemaker activity (Du et al. 2010); O'Grady et al. 2012). However, the magnitude of the change normalized by the average gastric fundal area, as well as the period of change was comparable, irrespective of MSG administration. Also, in agreement with the comparison in B, in the eight participants with accelerated gastric emptying with MSG, the gastric fundal motility [DM(A)] estimated from the standard deviation of the mean in the gastric fundal area in 40 images (P4 plane) was comparable for 60 min: 6.4 ± 2.3% without MSG, and 5.9 ± 4.4% with MSG in MS1 (*P *= 0.62); 9.7 ± 6.3% without MSG, and 7.5 ± 4.1% with MSG in MS3 (*P *= 0.42, *n* = 8). The results suggest that contractile gut activity other than the gastric fundus made a major contribution to the acceleration of gastric emptying with glutamate.

### Effects of MSG on duodenal motility

Gastric emptying is regulated by multiple factors, such as duodenal resistance as well as gastric contractility (Hunt and Stubbs 1975); Hunt et al. 1975). Therefore, duodenal motility needs to be assessed in those subjects who showed accelerated gastric emptying with MSG. Figure 3 shows enhanced duodenal motility by addition of MSG observed in the same participant in Figure 2. Segment images of the duodenum region successively acquired in MS3 are compared in A. Only small differences (noted by red and blue lines) were observed in the duodenum between images without MSG. On the other hand, contractile rings (green arrow head) occurred with MSG (see also Videos S5–S8). The Y‐shift of the center of gravity of the duodenal wall is plotted against time in B [DG_Y_(*t*)]: Greater shifts were observed with MSG during the propagation of contractile rings, agreeing well with the segment images in A.

### Numerical evaluation of duodenal motility

**Figure 7.**
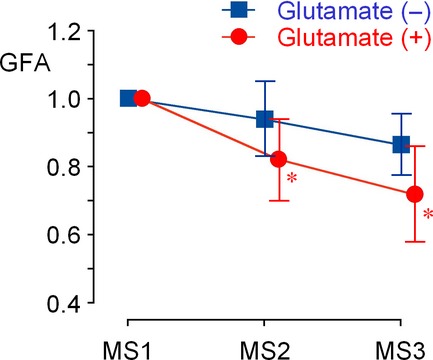
Changes in the gastric fundal area (GFA) in four of eight participants with positive responses to MSG and with duodenal wall sufficiently identified (means ± SD, *n* = 4), assuming the MS1 area as the unit. Each point represents the GFA averaged in 40 successive segment images in P4. Asterisks indicate *P *<**0.05, compared to the ingestion of liquid meal alone.

In four of the eight participants with positive responses to MSG (corresponding to those indicated by asterisks in Fig. 5), the contrast of the duodenal wall was sufficient to follow motility analyses. Figure 7 summarizes the acceleration of gastric emptying in these four participants. Without MSG, GFV decreased to 94.2 ± 12.2% after 30 min (MS2) and to 86.4 ± 8.9% after 60 min (MS3), whereas with MSG it decreased to 78.1 ± 3.5% after 30 min (MS2) and to 63.0 ± 3.1% after 60 min (MS3) (*P *<**0.05, *n* = 4, in MS2 and MS3).

**Figure 8.**
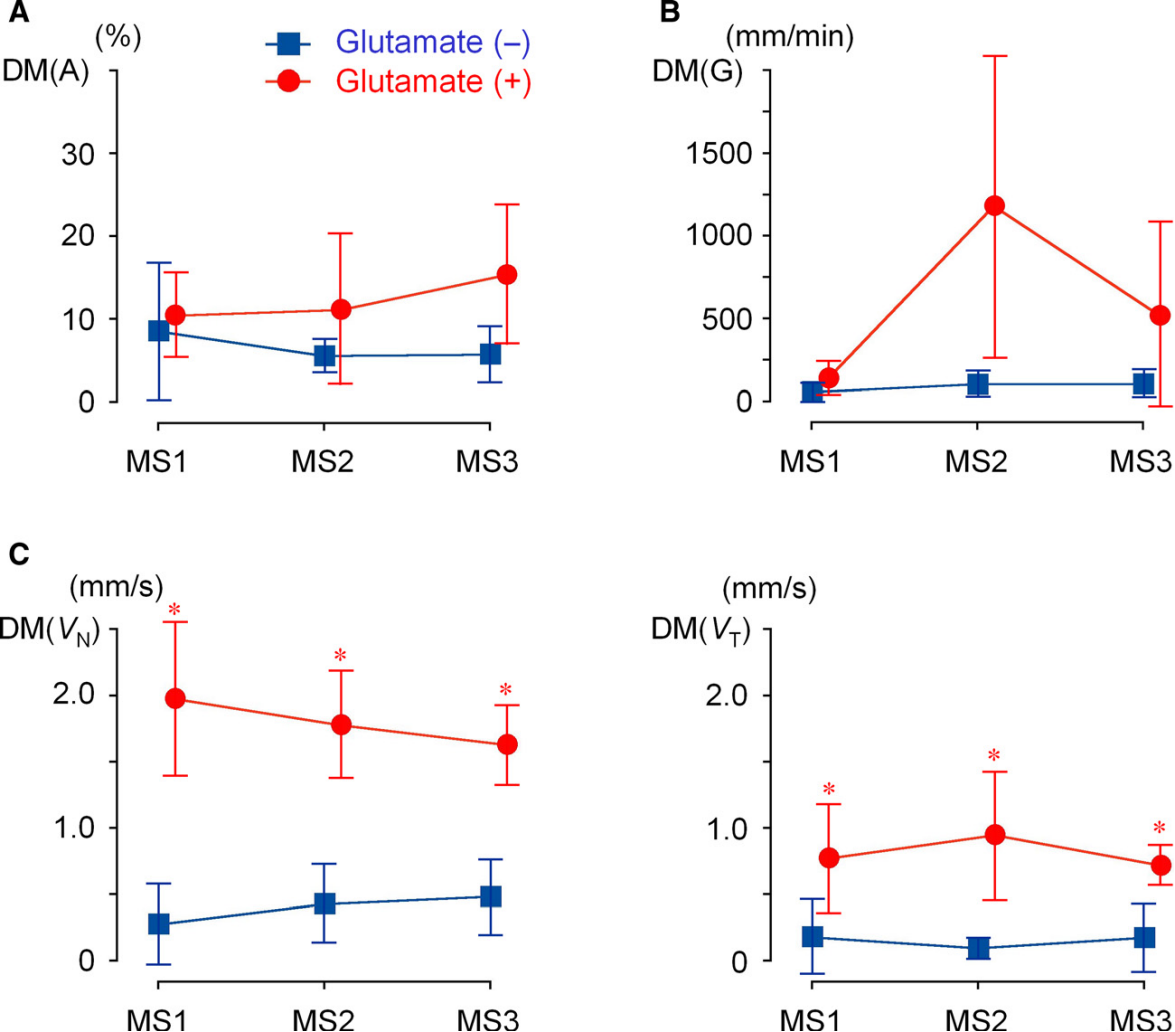
Numerical evaluation of duodenal motility in the four participants who positively responded to MSG with a sufficiently observable duodenal wall (*n* = 4). (A) SD of the mean DA in 40 images (DM(*A*)) (B) Changes in the center of gravity (DM(*G*)). (C) Mean velocity of duodenum wall motion along normal and tangential lines (DM(*V*_N_) and DM(*V*_T_)). Asterisks indicate *P *<**0.05, compared to the ingestion of liquid meal alone.

As indexes of duodenal motility, (1) the standard deviation of the mean in the duodenal area [DM(A)], (2) total shifts of the center of gravity [DM(G)], and (3) the mean velocities of the wall motion along the normal line and tangential line [DM(*V*_N_) and DM(*V*_T_)] were analyzed. Figure 8 shows changes in these three parameters evaluated from the duodenal segment images in the four participants with positive responses to MSG in gastric emptying. The addition of MSG caused sizeable increases in DM(A) and DM(G) after 30 and 60 min (MS2 and MS3); however, there was no statistically significant difference. On the other hand, both velocities DM(*V*_N_) and DM(*V*_T_) were significantly larger with MSG throughout the measurement (*P *<**0.05). Even immediately after the ingestion of liquid meal (MS1), DM(*V*_N_) and DM(*V*_T_) were 1.97 ± 0.59 mm/sec and 0.77 ± 0.42 mm/sec with MSG, but 0.27 ± 0.30 mm/sec and 0.18 ± 0.28 mm/sec without MSG. The increases in the velocities of duodenal wall motion with MSG agree well with the impression obtained from cine MRI (Supplemental Videos 5 and 7). Taken together, the results indicate that MSG added in liquid meal immediately facilitates duodenal motility probably through activation of glutamate receptors in the stomach, and that the velocity of wall shifts most appropriately reflects the excitatory effect of glutamate.

**Figure 9.**
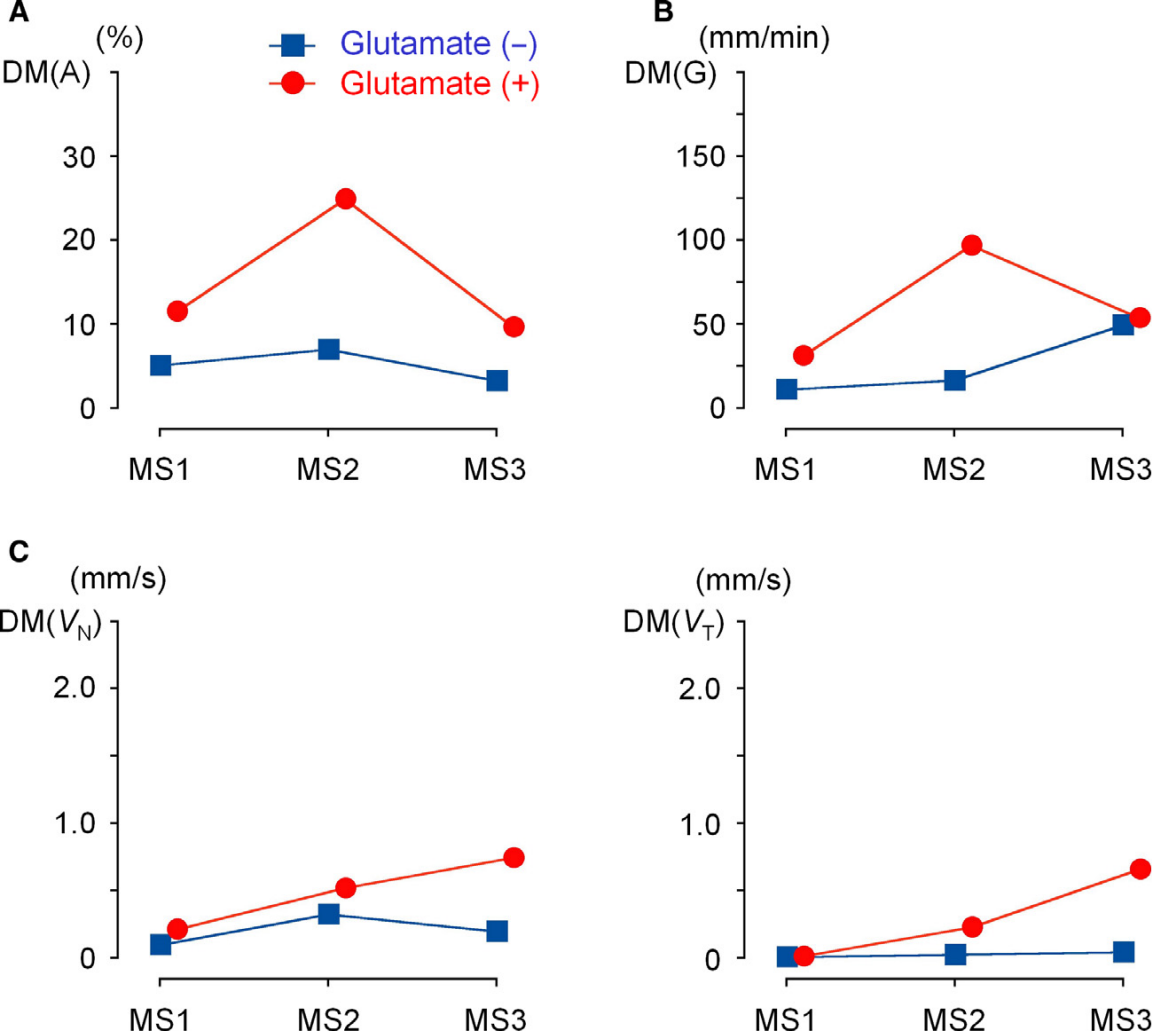
Numerical evaluation of duodenal motility in one of two participants with a negative response to MSG in gastric emptying. The duodenal wall was sufficiently observed. (A) SD of the mean DA in 40 images (DM(*A*)). (B) Changes in the center of gravity (DM(*G*)). (C) The mean velocity of duodenum wall motion along normal and tangential lines (DM(*V*_N_) and DM(*V*_T_)).

In one of two participants with negative responses to MSG, the contrast of the duodenal wall was also sufficient to follow motility analyses. The changes in DM(*A*), DM(*G*) and DM(*V*) are shown in Figure 9. The addition of MSG caused sizeable increases in all parameters of duodenal motility. For example, MSG increased DM(*V*_N_) by 1.5‐ to 2.5‐fold with MSG., although the absolute values of DM(*G*) and DM(*V*) were markedly smaller than those in participants with positive responses to MSG.

## Discussion

A rapid MRI scan of the abdominal cavity visualizes GI motility. In this study, the gastric residual volume (estimated from the serosal surface of the fundus area in multiple coronal planes) decreased to ~80% 60 min after ingestion of liquid meal (200 kcal in 200 mL), the rate of decrease being consistent with our previous study (Teramoto et al. 2012). Also, in eight of 10 participants, the addition of 0.5% MSG significantly accelerated the rate of decrease in the gastric fundus area (~73% after 60 min). These results reinforce a previous report (Zai et al. 2009) in which MSG promotes ^13^C‐labeled CO_2_ excretion upon ingestion of a protein‐rich liquid meal, suggesting accelerated gastric emptying. In addition, the present MRI measurements have shown how MSG actually enhances gut motility. Especially, enhanced duodenal contractions are a major contributor to gastric emptying.

Among three types of parameters, the velocities of duodenal wall motions (*V*_N_ and *V*_T_) most appropriately indicate the excitatory effect of glutamate on duodenal motility (Fig. 8C) upon the acceleration of gastric emptying. As shown in Figure 3A, MSG induced contraction rings during MRI measurements. It is considered that such contractile activity presumably caused the discrepancy between the parameters used to evaluate duodenal motility, that is, contractile rings in a lower part of the duodenum did not largely shift the center of gravity, but are surely detected as the velocity of wall motions. However, changes in the center of gravity and the GI area could be used to appropriately display motility in a series of segment images, depending upon the purpose of the study, such as changes in the trajectory of the center of gravity in comparison between the ingestion of liquid meal and water (Teramoto et al. 2012).

Two gating processes, such as oral feeding and gastric emptying (antroduodenal transport), regulate energy uptake of the human body. After the latter process, food is digested into small molecules, and nutrients are absorbed in the small intestine. The present MRI observations suggest that the effects of glutamate are comparable to those of several GI hormones and neurotransmitters related with the brain–gut interaction (Willie et al. 2001); Tsurugizawa et al. 2009); Janssen et al. 2011a),b; Karnani and Burdakov 2011); Nakayama 2011). As orexin and ghrelin affect both cephalic and gastric functions, l‐glutamate contained in food acts as umami taste to evoke appetite through glutamate receptors in the oral cavity, and also promotes antroduodenal transport by organizing gastric and duodenal contractions presumably by facilitating vagal activity (Niijima 1991); Niijima and Meguid 1995); Uneyama 2011). In the small intestine, absorbed nutrients suppress the secretion of these hormones which promote feeding (Janssen et al. 2011b). On the other hand, proteins are digested into individual amino acids including l‐glutamate, thereby increasing the amount of l‐glutamate in the small intestine. Among the amino acids, the special role of l‐glutamate is ascribed to the distinct distribution of amino acid receptors between the stomach and small intestine. Namely, only glutamate receptors are expressed in the stomach, whereas numerous amino acid receptors exist in the small intestine (Uneyama 2011). Thus, l‐glutamate alone causes the acceleration of gastroduodenal transport in the stomach, corresponding to its role as umami taste (appetizer) in the oral cavity.

In many Asian countries including Japan, Korea, and China, there is a growing influence of a highly aging population. For elderly people with eating disorders, tube feeding is a common clinical treatment. However, it has been pointed out that patients with such treatments often experience fever due to aspiration pneumonia (Mizock 2007). Indeed, our previous MRI measurements also demonstrated that gastric emptying is significantly slower in tube‐fed patients with fever (Teramoto et al. 2009). Thus, cotreatments with prokinetic agents are considered to reduce the risk of aspiration pneumonia. Previously, it was reported that serotonin 5‐HT_4_ receptor agonists and dopamine D_2_ receptor antagonists accelerate gastric emptying, accompanied by increases in proximal gastric tone and duodenal motility, respectively (Borovicka et al. 1999); Teramoto et al. 2012). However, the former agents cause a side effect, long QT syndrome (Chen et al. 2002). Also, it is well known that gut dysmotility disorders are frequently complicated in patients with parkinsonism, in which clinical treatments target dopamine D_2_ receptors in the brain (Kaneoke et al. 1995). Therefore, the administration of such chemicals needs careful consideration. In comparison, MSG may be applied more safely without considering such side effects in the heart and brain, although side effects on fluid secretion in the stomach need to be considered (Vasilevskaia et al. 1993); Bellisle 1998).

The prokinetic effect of MSG may be used as a special recipe. Indeed in Japan it looks popular to serve oily noodle soup with a secret ingredient of umami taste, containing l‐glutamate as the major component. The visceral sense of accelerated gastric emptying and duodenal motility may be unconsciously converted and integrated in our mind as a good taste through afferent nervous activity. Conversely, antagonistic food to glutamate receptors in the stomach, if any, may be used to improve overweight by suppressing antroduodenal transport and appetite.

In the majority of participants, the addition of MSG increased the rate of gastric emptying, but it was reduced in two of 10 participants. We thus checked the shape of the stomach in all coronal planes to see if liquid meal is mechanically trapped. However, no systematic difference in the shape of the stomach was observed between groups. These observations indicate that this limitation of MSG could allow it to be used as a prokinetic agent. The largely varied responses may be associated with personal differences in gut motility. For example, circadian rhythmicity, long‐term effects of a previous diet, and genetic mutations related with glutamate/serotonin signaling pathways possibly modulate the rate of gastric emptying (Ribeiro et al. 2011); Camilleri and Katzka 2012). Also, diabetes mellitus is a common popular disease, frequently complicated by gut motility disorders due to the impairment of enteric neurons and pacemaker cells (Vittal et al. 2007); Lin et al. 2010); Vanormelingen et al. 2013). Such pathological changes may have occurred in some participants. It is, therefore, preferable to individually evaluate the response to MSG. Furthermore, additional effects of MSG are considered to form personally different responses. Especially, in dogs and humans it has been reported that l‐glutamate‐mediated acid secretion may increase the risk of aspiration pneumonia, by increasing the gastric fluid volume (Vasilevskaia et al. 1993); Bellisle 1998). This effect may account for the discrepancy seen in Figure 9: MSG enhanced duodenal motility but slowed gastric emptying because MRI does not distinguish liquid meal and secreted fluid in the stomach. Thus, increased gastric secretion may mask the prokinetic effect of l‐glutamate. In such cases, additional ^13^C breath tests are recommended to precisely estimate the rate of gastric emptying. Alternatively, the discrepancy may be attributed to the limitation of the present evaluation of gut motility because the parameters used do not indicate the quality of gut motions, such as mixing, forward or backward transport of the content, etc.

In conclusion, this study provides rapid MRI observations that the addition of MSG may accelerate gastric emptying presumably due to a major contribution of enhanced duodenal motility in the majority of healthy subjects. This suggests the possible use of MSG as a prokinetic agent. However, more research is needed to confirm these findings. Also, we should consider the limitation namely that the responses to MSG varied between participants, and therefore it is preferable to individually assess the effects of MSG on gut motility. In this respect, rapid MRI combined with image analysis could be useful when reinforced by additional examinations such as the ^13^C breath test. Also, it is considered that numerical evaluation of gut motility using rapid MRI appears to have merit by being able to apply statistical analyses combining clinical data between hospitals and institutions.

## Acknowledgments

We thank Noriko Ishikawa and Kaori Oohashi (Kojin Hospital, Nagoya, Japan) for technical assistance.

## Conflict of Interest

Hidemi Teramoto, Hideto Yogo, Yuuta Nishimiya, Shinji Hori, and Shinsuke Nakayama have no conflicts of interest to declare. Toshiyasu Shimizu and Takashi Kosugi belong to R'Tech Corporation, which deals with segment software to analyze GI motility.
